# Tackling a global epidemic threat: Nipah surveillance in Bangladesh, 2006–2021

**DOI:** 10.1371/journal.pntd.0011617

**Published:** 2023-09-27

**Authors:** Syed Moinuddin Satter, Wasik Rahman Aquib, Sharmin Sultana, Ahmad Raihan Sharif, Arifa Nazneen, Muhammad Rashedul Alam, Ayesha Siddika, Fateha Akther Ema, Kamal Ibne Amin Chowdhury, Ahmed Nawsher Alam, Mahmudur Rahman, John D. Klena, Mohammed Ziaur Rahman, Sayera Banu, Tahmina Shirin, Joel M. Montgomery

**Affiliations:** 1 icddr,b, Dhaka, Bangladesh; 2 Institute of Epidemiology, Disease Control and Research (IEDCR), Dhaka, Bangladesh; 3 Global Health Development, EMPHNET, Dhaka, Bangladesh; 4 Viral Special Pathogens Branch, Centers for Disease Control and Prevention (CDC), Atlanta, Georgia, United States of America; NIAID Integrated Research Facility, UNITED STATES

## Abstract

Human Nipah virus (NiV) infection is an epidemic-prone disease and since the first recognized outbreak in Bangladesh in 2001, human infections have been detected almost every year. Due to its high case fatality rate and public health importance, a hospital-based Nipah sentinel surveillance was established in Bangladesh to promptly detect Nipah cases and respond to outbreaks at the earliest. The surveillance has been ongoing till present. The hospital-based sentinel surveillance was conducted at ten strategically chosen tertiary care hospitals distributed throughout Bangladesh. The surveillance staff ensured that routine screening, enrollment, data, and specimen collection from suspected Nipah cases were conducted daily. The specimens were then processed and transported to the reference laboratory of Institute of Epidemiology, Disease Control and Research (IEDCR) and icddr,b for confirmation of diagnosis through serology and molecular detection. From 2006 to 2021, through this hospital-based surveillance platform, 7,150 individuals were enrolled and tested for Nipah virus. Since 2001, 322 Nipah infections were identified in Bangladesh, 75% of whom were laboratory confirmed cases. Half of the reported cases were primary cases (162/322) having an established history of consuming raw date palm sap (DPS) or tari (fermented date palm sap) and 29% were infected through person-to-person transmission. Since the initiation of surveillance, 68% (218/322) of Nipah cases from Bangladesh have been identified from various parts of the country. Fever, vomiting, headache, fatigue, and increased salivation were the most common symptoms among enrolled Nipah patients. Till 2021, the overall case fatality rate of NiV infection in Bangladesh was 71%. This article emphasizes that the overall epidemiology of Nipah virus infection in Bangladesh has remained consistent throughout the years. This is the only systematic surveillance to detect human NiV infection globally. The findings from this surveillance have contributed to early detection of NiV cases in hospital settings, understanding of Nipah disease epidemiology, and have enabled timely public health interventions for prevention and containment of NiV infection. Although we still have much to learn regarding the transmission dynamics and risk factors of human NiV infection, surveillance has played a significant role in advancing our knowledge in this regard.

## Author summary

NiV is one of the most fatal emerging bat-borne zoonotic viruses, posing a threat to global health security. Since the first recognized outbreak in Bangladesh in 2001, human infections have been detected almost every year. In 2006, a hospital-based Nipah sentinel surveillance was established in Bangladesh for the early detection of cases and rapid response to outbreaks. Currently, this surveillance is running at ten strategically chosen public hospitals throughout Bangladesh. Till December 2021, a total of 322 Nipah cases have been reported in Bangladesh, 71% of whom has died during the course of infection. Throughout the years, fever, vomiting, headache, fatigue, and increased salivation have remained the most commonly observed symptoms. Half of the reported cases were primary cases (162/322) having an established history of consuming raw date palm sap (DPS) or tari, and 29% were infected through person-to-person transmission. Since the inception of surveillance, there has been a decrease in large outbreaks and a concurrent increase in sporadic Nipah case detection throughout the country. The overall epidemiology of Nipah virus infection in Bangladesh has remained consistent throughout the years. National Nipah surveillance of Bangladesh is the only global, systematic surveillance to detect human NiV infection. The findings from this surveillance have contributed to the early detection of Nipah cases, understanding of disease epidemiology, and implementation of timely public health interventions for the prevention and containment of human NiV infection.

## Introduction

The first outbreak of human NiV infection was reported in the Malaysia-Singapore peninsula between September 1998 and April 1999 [1,2]. Most cases from that event presented with respiratory illness followed by encephalitis-like symptoms, which proved fatal for 40% of the afflicted individuals [3,4]. Data from the Malaysian outbreak indicated that fruit bats (*Pteropus* spp.) were the natural host of this virus [5]. Pigs were infected due to eating fruits half-eaten by these bats, and human spillover occurred by handling infected pigs [6,7]. Soon after, in January 2001, an outbreak of a similar illness occurred in Siliguri, India, where 75% of the infected individuals died [8]. Two months later, in April 2001, a cluster of encephalitis cases was reported from Meherpur district of Bangladesh, where nine of the 13 infected individuals (69%) died [9]. At that time, testing facilities to confirm human NiV infection were limited to the United States Centers for Disease Control and Prevention (US, CDC) only. Through a retrospective investigation in 2003, the cluster identified in 2001 was recognized as the first outbreak of human NiV infection in Bangladesh [1,9]. IEDCR was the leading government institute responding to these events, in collaboration with icddr,b and with technical support from US, CDC.

Up to 2007, eight outbreaks were identified in Bangladesh from the central and north-western regions, almost all of which occurred during the winter season [5,10]. Out of the 104 Nipah cases detected between 2001 and 2006, more than 75% individuals died, and all the individuals either had a history of consuming raw DPS or close contact with individuals suffering from Nipah-like illness [5,11–13]. In Bangladesh, raw DPS is harvested during the winter (between October and April), and it is consumed raw, as a seasonal delicacy [14]. An event-based notification system followed by case or outbreak investigation was the standard practice in the early phases prior to the introduction of sentinel surveillance. Researchers suspected sporadic cases were under-reported since the data at that time was primarily derived from infections reported from known outbreaks [5]. Between 2004 and 2007, in addition to the patients linked to the known outbreaks, patients with encephalitis-like symptoms who sought treatment from the hospitals of that region were tested for the Nipah virus [5]. This revealed several sporadic NiV infections unrelated to the-then ongoing large-scale outbreaks [5]. It indicated that not all human NiV infections were being identified, emphasizing the need for a standardized surveillance system for the detection of Nipah cases [5].

In response to the outbreak and sporadic cases detected between 2001 and 2006, IEDCR and icddr,b introduced routine sentinel surveillance for human NiV infection in February 2006 at ten public hospitals with technical support from the US, CDC [15,16]. The goal of the surveillance was to facilitate early detection of encephalitis outbreaks, including those resulting from NiV, identification of associated risk factors, and understanding of transmission risks to enable timely public health interventions for prevention and containment [16]. The country’s first Nipah diagnostic laboratory was established at IEDCR in 2006. The surveillance officially came into effect at ten government hospitals in early 2007 [16]. The purpose of surveillance was to facilitate early detection of encephalitis outbreaks or clusters, including Nipah, identification of associated risk factors, and understanding of the transmission risks to enable timely public health interventions for prevention and containment [16]. The event-based notification was continued in the form of event-based surveillance, to complement active surveillance at the sentinel sites.

Over the past sixteen years, Nipah surveillance activities in Bangladesh have evolved substantially. Since the establishment of national surveillance, most NiV cases around the country have been detected through this system [11,16]. This article aims to describe the evolution and current approach of Nipah surveillance activities, the updated epidemiology of Nipah virus infection in Bangladesh, and recommend future actions and improvements to the existing system in order to detect and understand human-virus interaction more effectively.

## Methods

### Ethics statement

Informed written consent was obtained from the eligible cases or their legal guardian. This study protocol (PR-2005-026) was reviewed, and ethical approval was obtained from the ethical committee of icddr,b.

### Case finding strategy prior to initiation of surveillance

Prior to initiation of the surveillance, no formal/structural Nipah case reporting and detection strategy was in place. At that time, suspected Nipah cases were reported during active outbreaks or after the outbreak had occurred [17]. Broad context specific case definitions of suspected and probable Nipah cases were formulated and implemented as the outbreak investigations continued [17]. Two active case search strategies were adopted to detect Nipah cases. Firstly, a community-based/house-to-house case search in the known affected areas while the outbreak investigation was ongoing. Subsequently, an expanded case-finding activity was initiated at the tertiary healthcare facilities of the affected districts of Bangladesh, where all the hospitalized patients with fever and acute brain pathology were investigated [17]. This was jointly conducted by IEDCR and icddr,b.

### Surveillance sites and strategy

**Fig 1:**
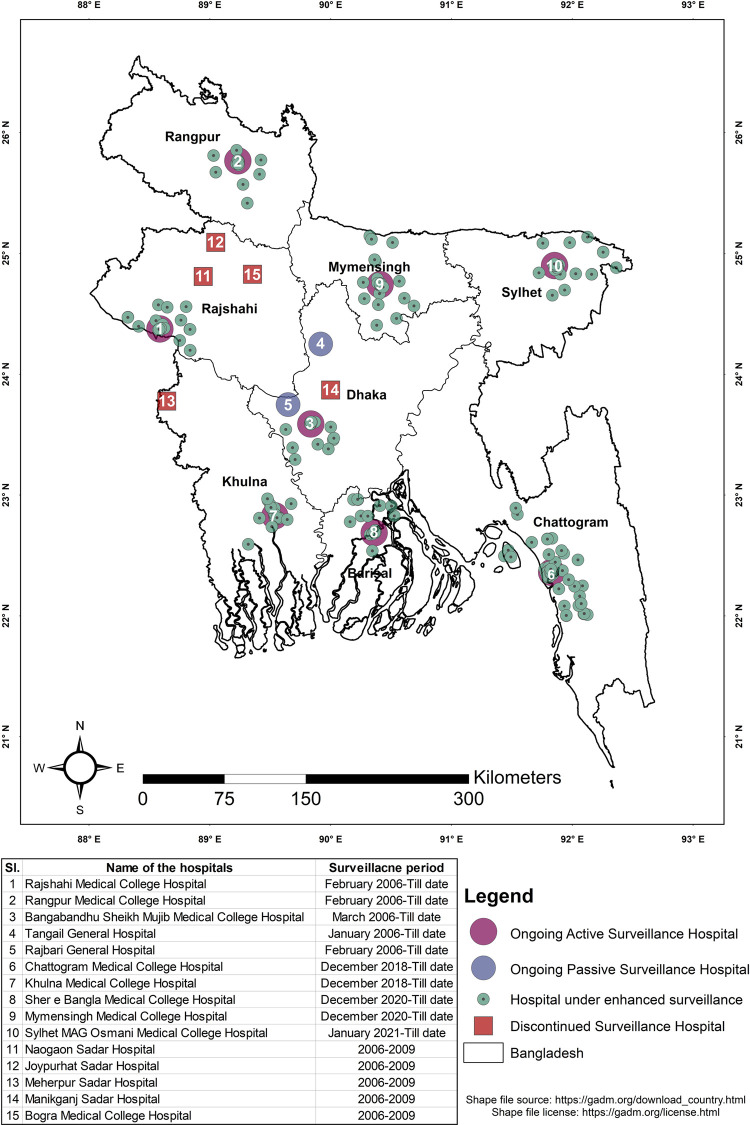
Historical information on Nipah surveillance sites, 2006 to 2021. Shape file source of the map: https://gadm.org/download_country.html Shape file license of the map: https://gadm.org/license.html.

Based on the experience from prior outbreaks and outbreak investigations, active surveillance was initiated in February 2006 at ten government hospitals across four administrative divisions of the country (Dhaka, Rajshahi, Rangpur, and Khulna), all within the north-west and central region (Fig 1). Initially, a cluster-based surveillance strategy was adopted at all sites [16]. The initial surveillance strategy at sentinel hospitals was to identify and investigate clusters of suspected meningo-encephalitis cases. Patients from adult medicine and pediatrics wards with “suspected Nipah encephalitis,” defined as fever (axillary temperature >38·5°C) with recently altered mental status or seizure or other neurological deficits suggestive of either diffuse or localized brain injury or “suspected Nipah pneumonitis,” defined as fever (axillary temperature >38·5°C) with severe shortness of breath and chest radiograph consistent with diffuse acute respiratory distress syndrome were screened [16]. Detailed addresses and telephone numbers of admitted patients were recorded, and they were cross-referenced to ascertain whether they were from the same community. To identify additional encephalitis cases, physicians asked admitted cases and/or their caregivers about other ill persons or recent deaths with similar symptoms in their communities who did not report to the hospitals or died during the initial phase of their illness. An encephalitis/pneumonitis cluster was defined as more than two suspected Nipah-encephalitis/pneumonitis cases aged above five years of age, living within 30 minutes walking distance of each other, who developed illness within three weeks. The cut-off of 3 weeks was decided based on the incubation period and transmissibility of NiV [17].

A year later, four district hospitals were converted to passive surveillance sites as few cases of encephalitis were reported from those sites [16]. Passive sites only reported to surveillance authorities if they identified an unusually large number of suspected meningo-encephalitis/viral pneumonitis cases. In contrast, active sites reported the total number of suspected Nipah encephalitis/pneumonitis cases admitted every month and notified regarding clusters as soon as they were identified [16]. Based on the seasonality of Nipah infection within the country, in 2007, a case-based component was introduced in the surveillance. Under this component, blood, oral/throat swab, and cerebrospinal fluid (CSF) were collected from all suspected Nipah encephalitis/pneumonitis cases admitted between January and March of each year (Table 1). This was started at Faridpur Medical College Hospital, Rajshahi Medical College Hospital in 2007, and Rangpur Medical College Hospital in 2008. The justification for choosing these hospitals was the significant number of cases reported from respective catchment areas in previous years. In 2009, surveillance was discontinued at four passive sites (Jaypurhat, Naogaon, Meherpur, and Manikganj district hospital) and one active surveillance site (Bogura Medical College Hospital) (Fig 1). The decision was based on the fewer number of suspected encephalitis cases reported from these sites since the initiation of the surveillance. From 2009 to 2018, covering all the Nipah-prone regions of the country, active surveillance was carried out at three hospitals (Faridpur, Rajshahi, and Rangpur Medical College Hospital), and passive surveillance was carried out at two (Rajbari and Tangail General Hospital) (Fig 1). During this period, case and cluster-based surveillance were carried out throughout the year at active sites. For passive sites, the strategy remained unchanged.

**Table 1:** Surveillance case definitions of human NiV infection.

Case definition	Criteria
Nipah like illness (Screening case definition)	Fever (axillary temperature >38.5°C) or history of raised temperature prior to hospitalization **AND** any sign/symptom of brain pathology **OR** Fever (axillary temperature >38.5°C) or history of raised temperature prior to hospitalization **AND** any sign/symptom of lung pathology
Suspected Nipah encephalitis	Fever or history of fever (axillary temperature >38.5°C) **AND** evidence of acute brain pathology (e.g., altered mental status, new-onset seizures, or new neurological deficit either diffuse or localized to the brain)
Suspected Nipah pneumonitis	Onset of illness within the last seven days **AND** fever or history of fever (axillary temperature >38.5°C) **AND** severe shortness of breath (i.e., dyspnea that prevents the patient from walking unassisted for not more than ten steps) **AND** chest radiograph consistent with diffuse acute respiratory distress syndrome
Probable Nipah infection	History of Nipah encephalitis or pneumonitis **AND** established epidemiological link with at least one confirmed Nipah case but the absence of laboratory confirmation due to the patient’s death
Confirmed Nipah infection	Laboratory confirmation of the infection; either by identification of anti-Nipah IgM via ELISA (Enzyme-linked immunosorbent assay) OR through identification of viral particles by qRT-PCR (quantitative reverse transcription polymerase chain reaction) with or without a history of Nipah encephalitis/pneumonitis

Data from this surveillance suggested that from 2007 to 2014, almost half of the Nipah outbreaks were not being reported as the patients lived in areas distant from the surveillance hospitals [18]. Based on this finding, in 2018, the surveillance strategy was revised, and the authority decided to bring the whole country under active surveillance with more focus on finding cases. In 2018, as part of this initiative, surveillance was expanded to include two additional tertiary care hospitals: Khulna Medical College Hospital and Chattogram Medical College Hospital. That same year, considering the load of patients, and to ensure justified utilization of resources and optimum functionality of surveillance a two-tier case-finding approach was adopted. An initial screening was conducted for Nipah-like illness, with a wider case definition, followed by a thorough eligibility assessment as per the Nipah encephalitis/pneumonitis case definition (Table 1). Surveillance case definitions for both suspected Nipah encephalitis and pneumonitis were further redefined as well (Table 1).

Two years later, in 2020, three additional sites were included as active surveillance sites (Mymensingh Medical College Hospital, Sylhet Medical College Hospital, and Barisal Medical College Hospital). Thus, all eight administrative divisions were brought under surveillance for NiV infection.

### Nipah season

In Bangladesh, all the Nipah outbreaks coincided with the DPS harvesting season (mid-October to early April). Therefore, for operational purposes, this period was designated as the Nipah season. At the inception of surveillance, December to March was considered Nipah season. In 2022, Upon considering the availability of raw DPS and the number of Nipah cases reported in April, December to the End of April was declared as Nipah season in Bangladesh. During these months, communication with surveillance hospital authority, surveillance activities at the hospital level, transportation, and testing of samples are reinforced systematically.

### Evolution of surveillance activity

At the active surveillance sites, three surveillance physicians (two from medicine and one from the pediatrics department) of respective hospitals and two field staff carried out the surveillance together. At the passive surveillance sites, only one surveillance physician from the respective hospital was in charge of the activities. Throughout the year, surveillance staff were posted at the medicine and pediatrics wards of active surveillance sites who screened the admitted patients for symptoms of NiV infection. Until September 2020, active surveillance was conducted for eight hours daily from 8 am to 4 pm. Since then, daily surveillance activity has been extended up to 14 hours (8 am to 10 pm) to increase case enrollment during the late hours. This was in response to the admission of several suspected Nipah cases during late hours who were found to be positive for NiV the following day.

At each sentinel surveillance site, every day (except for Friday and government holidays), from 8 am to 10 pm, surveillance field staff screened hospitalized patients from the respective wards as per the screening criteria (Table 1). The screening process included a logbook review of medicine and pediatric wards, communication with the physician in charge, and an informal interview with the patient/patient’s attendant in search of symptoms suggestive of Nipah infection (Table 1). Upon identifying individuals with symptoms suggestive of Nipah infection, the surveillance physicians were notified. These physicians then performed a thorough eligibility assessment of the patients, adhering to the suspected Nipah case definition criteria. (Table 1). Upon matching the case definition, suspected Nipah cases were then enrolled, and informed written consent was obtained. Demographic, clinical, and exposure history were recorded through a structured questionnaire using an electronic device. The surveillance staff collected serum, throat swabs (TS), and CSF by maintaining all biosafety measures.

Adhering to all biosafety and security standard operating procedures, surveillance staff processed the collected samples in the biosafety hood (BSL 2 plus) set up by the surveillance authority at the respective surveillance sites. The sample vials were labeled with a unique ID and shipped to the virology laboratory of IEDCR and icddr,b, Dhaka in a liquid nitrogen dry shipper.

Samples were transported from sentinel sites to Dhaka every three days to facilitate early case detection during the ‘Nipah season.’ In cases where a suspected NiV patient demonstrated a clear epidemiological linkage to NiV, such as a history of consuming raw DPS or Tari, a history of consuming fruits partially eaten by an animal (possibly a bat), contact with a sick domestic or wild animal, contact with patients exhibiting symptoms compatible with NiV infection, from any place of the county, their samples were transported to Dhaka for testing within 24 hours of collection. For the remaining period of the year (May to November), samples are transported every fifteen to seventeen days based on the cryogenic temperature of liquid nitrogen dry shippers.

After receiving the samples at icddr,b, samples were inspected, cross-checked, and organized for distribution. During the Nipah season, serum samples were sent to IEDCR for serological evaluation against Nipah virus infection (anti-Nipah IgM and IgG). Simultaneously, qRT-PCR was conducted on the throat swab and serum samples collected in lysis buffer at the One Health laboratory of icddr,b, to observe the presence of viral nucleic acid.

At IEDCR, ELISA for Anti NiV IgG and IgM were carried out. The ELISA assay was carried out according to the procedure designed and recommended by CDC, Atlanta [12,19,20]. In brief, for anti-Nipah IgM, a microtiter plate (Corning, Glendale, AZ, USA) was coated with anti-human IgM antibody (1:500, KPL Antibodies & Conjugates (Seracare, Milford, MA, USA) [21]. Processed sera (heat inactivated and chemically treated) collected from patients were added to the appropriate wells in different dilutions (1: 100, 400, 1600 & 6400) [21]. After incubation at 37°C, CDC-provided Nipah cell slurry (inactivated NiV culture with Vero E6 cell line) mixed with normal human sera (provided by CDC, Atlanta), and slurry of control Vero E6 cell line was added according to the procedure as described previously and was incubated at 37°C [21]. Hyper-immune mouse ascitic fluid (HMAF, an ascitic fluid obtained from mouse which was immunized by NiV) consisting of Nipah antibody (dilution 1:4000, provided by CDC, Atlanta) was then added. HRP conjugated anti-mouse IgG, IgM (Thermo Fisher Scientific, Waltham, MA, USA) at a dilution of 1:8000 was added. 2,2′-azino-di (3-ethylbenzthiazoline-6-sulfonate) (ABTS) (Seracare, Milford, MA, USA) was used as the substrate and incubated for 30 min. The optical density (OD) of each well was measured at 414 nm using Epoch2 microplate reader (BioTek, Santa Clara, CA, USA).

For the detection of anti-Nipah IgG human antibodies, a microtiter plate (Corning, Glendale, AZ, USA) was coated with CDC-provided Nipah cell lysates of NiV culture with the Vero E6 cell line in PBS at 1:1000 dilution and incubated overnight at 4°C. Processed human serum (heat inactivated and chemically treated) was added to the plate using the same dilutions as mentioned above and kept at 37°C for an hour. HRP conjugated mouse anti-human IgG (Accurate Chemical & Scientific Corporation, Westbury Ave, Carle Place, NY, USA) at a concentration of 1:4000 was added after incubation to the wells, and ABTS was used as the substrate. The color development and OD measuring process were the same as IgM detection. The result was calculated as described previously [21].

For real-time RT-PCR, viral nucleic acid was extracted from 200 μL of serum or swab samples collected in lysis buffer (NucliSENS easyMag, bioMerieux Inc., Rodolphe St., Durham, NC, USA) using InviMag Virus DNA/RNA Mini Kit (INVITEK Molecular, Berlin-Buch GmbH, Germany) on Kingfisher Flex 96 (Thermo Fisher Scientific Inc., Waltham, MA, USA) automated nucleic acid extraction system according to the manufacturer’s instructions. The nucleic acids were eluted in 120 μL of elution buffer and stored at −80°C. Five microliters of extracted nucleic acid were used as a template for 25 μL of one-step RT-PCR reaction volume. Initially, *Taq*Man PCR assay was used to screen NiV RNA using NiV N gene-specific primers and probe, as described by Lo et al. [19]. One-step RT-PCR reactions were performed using the AgPath-ID one-step RT-PCR kit. In brief, reverse transcription was carried out for 10 min at 50°C followed by initial denaturation at 95°C for 3 min, and PCR was conducted for 45 cycles at 95°C for 15 s and 60°C for 1 minute. The results were analyzed using CFX Maestro Software 1.1 version (Bio-Rad Laboratories, Inc., Hercules, CA, USA) for quality of the amplification curve and determination of cycle threshold (Ct) values. The samples were considered positive if Ct ≤ 37 and of the good amplification curves.

From May to November, the throat swabs in lysis buffer were evaluated for evidence of NiV by qRT-PCR using a pooling method (five samples are combined in one tube). The protocol was to retest individual samples from a pool, if the pooled sample was positive for NiV qRT-PCR. All collected samples were stored at -80°C freezer in icddr,b. An aliquot from all positive test samples and 10% negative samples were routinely shipped to the Viral Special Pathogens Branch (VSPV) of the US CDC, Atlanta, for quality control.

From December to April, surveillance activity was extended to the sub-district government and private health care facilities, adjacent to the sentinel surveillance sites (Fig 1). This activity is designated as ‘Nipah enhanced surveillance’ and it was first introduced in 2016 to sensitize the health facilities to detect more cases. Posters with awareness messages, meetings with respective government and private stakeholders, hotlines, and logistic support have been provided under this activity. In 2021, 455 healthcare facilities were included under enhanced surveillance activity.

### Confirmed case notification and investigation

If an enrolled patient was confirmed to have NiV infection, the hospital authority was notified immediately, and they took standardized steps to isolate the patient to reduce the possibility of human-to-human transmission. Identification of a single laboratory-confirmed Nipah case is considered an outbreak. Upon confirmation of a NiV infection, an outbreak response team consisting of experts from IEDCR and icddr,b was immediately dispatched to the site to conduct a case investigation. Nipah virus infection is a bat-borne disease and it is known to affect domestic animals, primarily pigs. Intersectoral, coordinated epidemiological and field investigations and cross-checking of samples at both human and animal laboratories is essential in rapid control of any outbreak. Outbreak investigations for Nipah virus infection in Bangladesh have always been conducted with One health approach with experts in epidemiology, anthropology, sociology, and veterinary sciences.

This team initiated all necessary steps to reduce transmission and to contain the outbreak. This group of experts performed contact tracing, exploration of the epidemiological link of virus spillover, and collected samples from every individual with possible exposure to the confirmed case. Raising awareness of NiV among the population of the affected community was also the responsibility of this team. Meanwhile, the surveillance staff continued collecting throat swab samples from the Nipah patient daily until two consecutive PCR tests were negative and serum samples were collected every fourth day from enrollment until two consecutive PCR tests were negative.

*Data analysis*: We reported the number of suspected Nipah patients hospitalized and tested and the number of Nipah-positive specimens identified before and during the surveillance period by a year. Surveillance years were defined as January to December of the same year. We also reported age, sex, demographic distribution, clinical information, and detailed exposure history of Nipah-positive cases using standard questionnaires.

## Results

### Geographical distribution of Nipah cases

From April 2001 to December 2021, 322 human NiV cases have been reported in Bangladesh, including 241 (75%) confirmed and 81 (25%) probable cases (Table 2). Nipah cases are distributed over 33 districts of Bangladesh, and out of the eight administrative divisions, the majority of cases were reported from four (Dhaka, Khulna, Rajshahi, and Rangpur). Prior to the establishment of the surveillance (till 2006), 104 cases were detected nationwide, two-third (68%) of which were from the Dhaka division (Table 2). Since the establishment of surveillance in 2006, 218 cases have been detected nationwide through this surveillance system, which is more than two-thirds of the total cases reported to date (Table 2). Before establishing the surveillance, less than 10% of NiV cases were sporadic, whereas, after the initiation of surveillance, almost 40% of the detected cases were sporadic.

**Table 2:** Geographical distribution of Nipah cases in Bangladesh from April 2001 to December 2021 (N = 322).

Division & District	2001	2002	2003	2004	2005	2006	2007	2008	2009	2010	2011	2012	2013	2014	2015	2016	2017	2018	2019	2020	2021	Grand Total
**Barishal Division**																						**1**
Jhalokathi																				1		1
**Chattogram Division**																						**1**
Comilla											1											1
**Dhaka Division**																						**147**
Dhaka				1																		1
Faridpur				37				1		12		1	1	8	3		2	1		5		71
Gopalganj				1						2		1	1	1	1					1		8
Madaripur										1				3	2							6
Manikgonj				6				4					6	1								17
Rajbari				14				6	1	2	2	1	2		1						1	30
Sariatpur														2								2
Tangail					12																	12
**Khulna Division**																						**36**
Chuadanga														1								1
Jhinaidah													1	1								2
Khulna														1								1
Kushtia							8			1			2	1								12
Magura													1	4	1							6
Meherpur	13																					13
Norail														1								1
**Mymensingh Division**																						**2**
Mymensingh													2									2
**Rajshahi Division**																						**53**
Bogra												1						2				3
Chapai-nawabgonj														1								1
Naogaon			12	2			1						3	1	4				1		1	25
Natore				1			1					1	2	1	1				1			8
Pabna							1						2				1					4
Rajshahi												4	2	3	1			1	1			12
**Rangpur Division**																						**82**
Dinajpur									1		6	1		1								9
Gaibandha													1									1
Joypurhat				4								6										10
Kurigram											1		1		1							3
Lalmonirhat											23		1									24
Nilphamari									1		1		3	1								6
Panchagar														1								1
Rangpur				1					1		9	1		4								16
Thakurgaon							7												5			12
**Grand Total**	**13**	**0**	**12**	**67**	**12**	**0**	**18**	**11**	**4**	**18**	**43**	**17**	**31**	**37**	**15**	**0**	**3**	**4**	**8**	**7**	**2**	**322**

**Fig 2:**
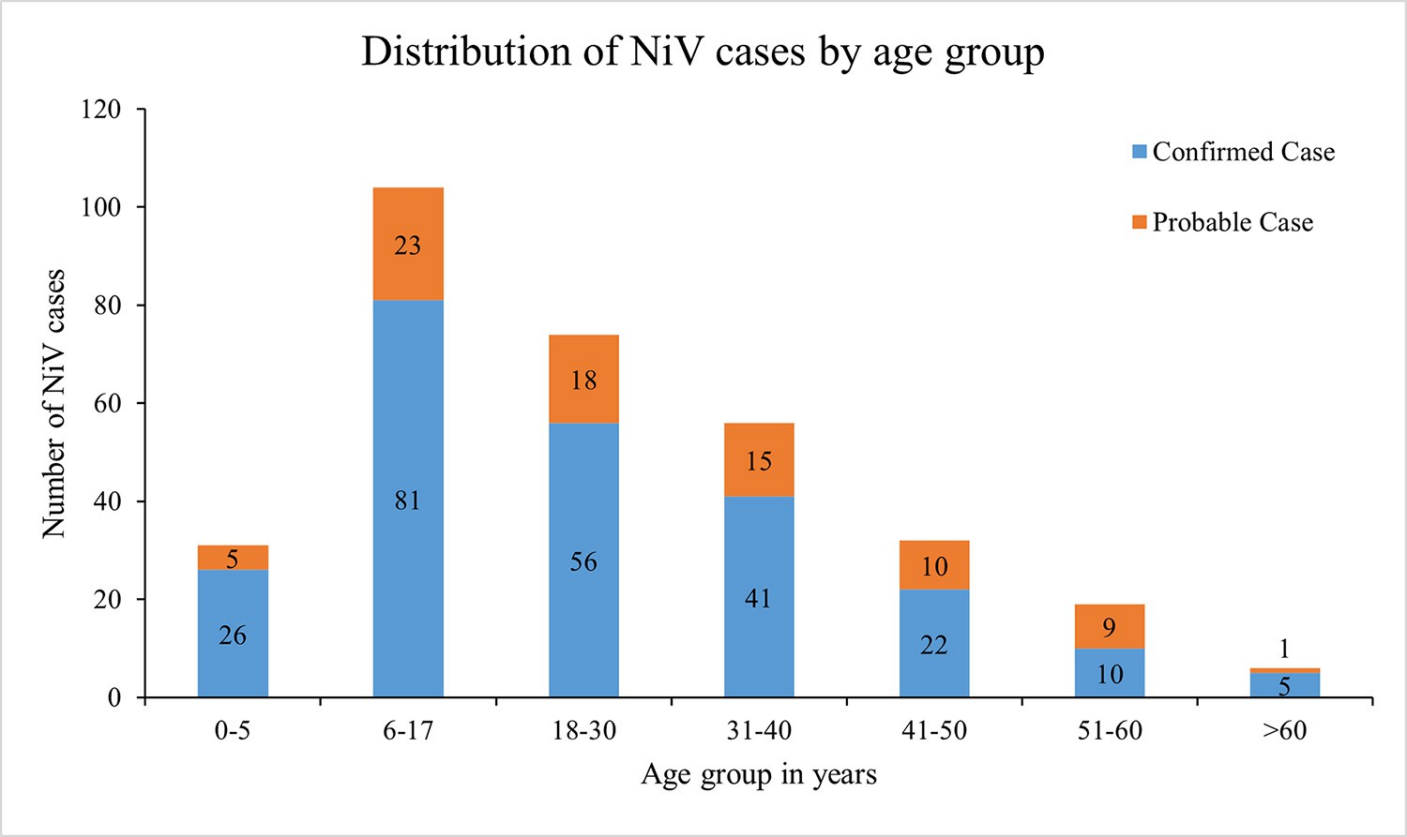
Age distribution of NiV patients (N = 322) who were identified from April 2001 to December 2021.

More than half (55%) of the NiV cases were between the age of 6 and 30 years (Fig 2). The median age of the NiV cases was 24 years (IQR, 10–35 years) (Table 3). 62% (201/322) of NiV-cases were male.

**Table 3:** Characteristics of NiV patients, including clinical symptoms and signs, exposure, and outcome of NiV infections reported from Bangladesh between April 2001 to December 2021.

Trait	Frequency (n)	Percentage (%)
**Exposure history (N = 322)**		
H/O Date palm sap consumption	156	48
History of close contact with Nipah case	93	29
H/O Tari consumption	6	2
Unidentified/unconfirmed source of infection	67	21
**General clinical findings**		
Fever (N = 322)	320	99
Muscle pain (N = 299)	113	37
Joint pain (N = 286)	62	22
Vomiting (N = 317)	176	56
Diarrhea (N = 318)	67	21
Headache (N = 308)	208	68
Fatigue/weakness (N = 316)	231	73
Increased salivation (N = 46)	44	96
**Clinical findings related to the respiratory system**		
Cough (N = 314)	156	50
Difficulty in breathing (N = 315)	183	58
ARDS (N = 126)	5	4
**Clinical findings related to the nervous system**		
Drowsiness (N = 293)	151	52
Personality change/altered mental status (N = 287)	117	41
Irritability/restlessness (N = 294)	103	35
Convulsion (N = 317)	132	42
Unconsciousness (N = 318)	255	80
**Outcome following initial hospitalization (death)**	228	71
**Lowest recorded GCS score during hospitalization (N = 66)**		
<3	9	14
3–8	8	12
9–12	5	8
13–15	44	67
**Time to hospitalization after symptom onset (N = 285)**		
≤ 3 days	108	38
4–5 days	96	34
>5 days	81	28
**Hospital stay (N = 67)**		
≤ 5 days	22	33
6–10 days	12	18
11–15 days	13	19
>15 days	20	30

*Although the total number of cases is 322, patient data of all the variables for all 322 cases are not available. Therefore, the denominator (N) or the number of Nipah cases asked/inquired about that individual variable is mentioned on the side. A total of 104 (32%) cases were reported before the establishment of surveillance, and 81 (25%) were probable cases. From these individuals, we do not have data against all the variables.

### Clinical features and risk factors

Half of the Nipah cases were due to primary infection and had a history of consuming raw DPS or tari prior to their onset of symptoms. The majority of the remaining cases had close contact with at least one laboratory-confirmed Nipah patient. However, no exposure history could be identified for at least 21% of cases (Table 3). The most commonly reported general clinical symptoms were fever, headache, fatigue or weakness, and vomiting, while unconsciousness and drowsiness were the predominant features of neurological involvement (Table 3). Even though information about increased salivation could be obtained from only a small number of Nipah cases, it was still highly prevalent among those affected. (Table 3).

Out of the 139 NiV cases reported since the initiation of surveillance, we were able to record a Glasgow Coma Scale (GCS) score from 66 individuals. Among them, 14% (9/66) of cases had decreased scores (less than 3), whereas more than two-thirds had near-normal [13–15] GCS scores while they were hospitalized (Table 3).

**Fig 3:**
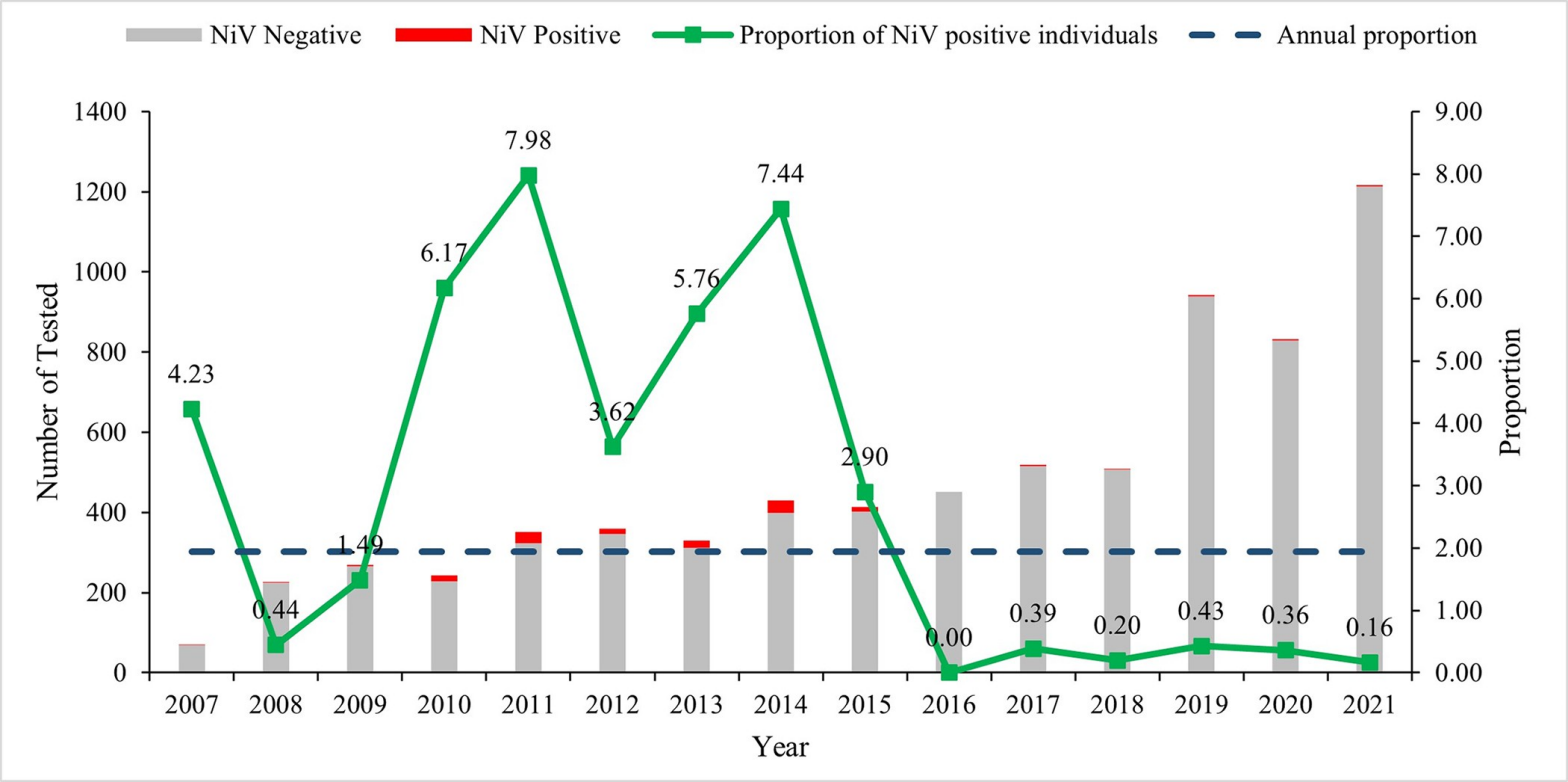
Distribution of individuals tested for NiV infection at ten sentinel hospitals in Bangladesh, January 2007 to December 2021. The red bars represent the number of NiV-positive persons (n = 139), and the grey bars indicate the number of NiV-negative individuals (n = 7011). The small green squares signify the proportion (%) of Nipah cases detected in the calendar year. The horizontal dark blue line implies the overall annual proportion of 1.94%. * In 2007, active sites started reporting the total number of suspected Nipah encephalitis/pneumonitis cases admitted every month.

During the surveillance period, from January 2007 to December 2021, 15,676 suspected Nipah cases were screened in the medicine and pediatric ward of surveillance hospitals. Surveillance staff enrolled 46% (7150/15676) of the suspected individuals and tested them for NiV. Of those suspect NiV cases (n = 7150) cases tested, 139 (2%) were positive for NiV (Fig 3). Test positivity remains the highest in the Faridpur region (4.4%) (Table 2). Since the inception of the surveillance, annual test positivity has remained relatively high till 2015. Since then, test positivity has remained very low at ⁓ 1% (Fig 3).

72% (204/285) of the Nipah cases were admitted within five days of symptom onset. Among all the NiV cases detected in Bangladesh till December 2021, 71% had a fatal outcome (Table 3). The annual detection of NiV cases and their fatality has fluctuated since its emergence in Bangladesh. Since 2001, Nipah cases were not reported in 2002, 2006, and 2016 (Fig 3).

## Discussion

Setting up and maintaining such surveillance throughout the year is challenging; similar initiative in other Nipah-affected countries is hard to find. To our knowledge, this is the only hospital-based active sentinel surveillance for human Nipah virus infection being conducted in any country, worldwide. Although NiV surveillance exists in several Nipah-prone countries of southeast Asia, none focus on human disease to such an extent. Malaysia, the first country to report human NiV infection, adopted a one-health approach for NiV surveillance with a significant human component [22]. Their event-based surveillance structure was focused on human cases to understand the disease epidemiology, sequelae, and strategies to protect the high-risk group [22]. Whereas In Bangladesh, the surveillance strategy for NiV includes year-round monitoring of human infections across all operational divisions of the country, covering a significant segment of the population. Thailand has also adopted a one-health approach for NiV surveillance focused on bats, pigs, and humans. However, their surveillance is concentrated in a single location where previous instances of NiV infection were detected in bats [23]. Their strategy of testing stored samples and healthy volunteers for NiV infection differs significantly from approach adopted in Bangladesh. Despite reporting four outbreaks of human NiV infection since 2001, India is implementing systematic NiV surveillance only among the bat population [24–29].

In several countries around the world, syndromic surveillance for meningo-encephalitis has been carried out. Such type of surveillance is methodologically similar to the national Nipah surveillance of Bangladesh, but none are solely focused on the NiV [30–33]. In Bangladesh, two similar surveillances were initiated around the same timeline to detect Japanese Encephalitis (JE) infection and NiV infection in the human population [34,35]. The case definitions and methodology of both these surveillances were very similar to the ongoing Nipah surveillance, but as the JE virus was not considered to have the potential to cause large-scale outbreaks/epidemics, the surveillance was carried out on a small scale and did not have an outbreak response component [34,35].

This hospital-based sentinel surveillance has been the key component in Nipah case detection in Bangladesh. The One Health approach adopted during the Nipah outbreak response helped identify the source of the outbreak and to devise strategies to prevent its further spread. It also highlighted the importance of the interconnectedness of human, animal, and environmental health.

The only method of detecting Nipah cases in Bangladesh before introducing systematic surveillance was through outbreak investigations and case finding in the affected area during/after the outbreak investigation [5]. Up to 2007, 48% (59/122) of Nipah cases could be confirmed through laboratory investigation. From 2007 to December 2021, 200 Nipah cases have been identified throughout the country, of which 80% (160/200) have been confirmed by laboratory testing [5]. This indicates an increase in Nipah case confirmation as well as the performance of the surveillance system in place. Besides, a significant increase in the detection of sporadic cases after 2007 (40% in comparison to 10% before the initiation of surveillance) is one of the significant achievements of this surveillance over the years. Nevertheless, the data from this very surveillance concluded that, from 2007 to 2014, almost half of the outbreaks were missed as the patients did not reach the sentinel surveillance hospitals [18]. The sentinel surveillance strategy was soon modified to cover more population and better detect cases from surveillance sites.

The age and gender distribution of the NiV cases and the prevalence of human-to-human transmission are unlike the findings from Malaysia but similar to most of the outbreaks from India [22,24,26,29,36]. The primary reasons for this variation can be attributed to the spillover mode and the subsequent transmission between animals and humans, as these play crucial roles in shaping the observed differences. Pig handlers, mainly working-age males were the primary populations affected in the Malaysia-Singapore outbreak; human-to-human transmission was not observed as a primary transmission route [22]. However, in Bangladesh and India, raw DPS/Tari consumption is the most common source of spillover (except the latest outbreak in Kerala, India), which often take place during family or social gatherings [37,38]. Due to the close-knit societal structure of the Indian sub-continent, visiting and taking care of sick individuals is the go-to family practice, irrespective of the threat of transmission. These contribute to the broader age range and gender distribution of NiV infections and the high prevalence of human-to-human transmission in this region [39]. Over the years, the age and gender distribution pattern of Nipah cases in Bangladesh remained almost the same [5,11].

Fever has always been the most prevalent symptom of NiV infection, but during earlier years, fever was mainly associated with altered mental status (82/91) [17]. Over the years, fever, followed by unconsciousness, has become the most prevalent clinical feature among Nipah patients. Other symptoms such as weakness, headache, cough, and difficulty breathing were present in most cases consistently throughout the years [5,11,17]. In recent cases, an increase in salivation (44/46) has been reported frequently.

Neurological symptoms were more common among the NiV cases in Bangladesh, resembling the findings from India, Malaysia, and Singapore [29]. Respiratory complaints were reported mainly from probable cases through the relatives of the deceased, which would have possibly been subject to recall bias.

Unlike Malaysia, a significant portion of the NiV infections in Bangladesh can be explained by transmissions linked to close contact with NiV cases [9,12,13,22,40,41]. Data from all four outbreaks in India bring forward strong evidence of transmission through person-to-person contact, similar to the findings from Bangladesh [24,26,29,36].

The overall case fatality rate (CFR) from Bangladesh now stands at 71%, less than the CFR of India and the Philippines but much higher than CFR among the cases from Malaysia and Singapore [42]. Low CFR from the Malaysia-Singapore outbreak could be due to the availability of necessary healthcare facilities, including critical patient care. In contrast, the absence of systematic Human NiV surveillance in India and the rest of the countries may have led to delayed detection with a low probability of survival, resulting in high CFR.

The findings presented in this paper indicate that, over the past 20 years, there has not been a remarkable change in the epidemiology of NiV in terms of spillover, transmission, clinical presentation, and fatality [5,11,17].

Despite having such a robust surveillance platform, most of the knowledge regarding this virus and its pathogenicity in humans is yet to be discovered. This is mainly attributed to its high fatality in humans. The interaction of the human immune system and the NiV is one of the critical issues to explore in the future, keeping in mind the potential of the large-scale outbreak and the newfound drive to develop vaccines and therapeutics against Nipah.

### Limitation

As this surveillance has been ongoing for 16 years, we have observed several limitations over the years. Maintaining a robust sentinel surveillance of such kind requires a continuous collaborative effort involving the government, central and local healthcare authorities, local administration, and the population at risk. As this virus conventionally spills over through the consumption of a local delicacy, it has been particularly challenging to convince people to avoid it. Moreover, case detection has not been consistent over the years; hence, awareness regarding this deadly virus has not prevailed consistently among the at-risk population, including healthcare workers. Rapid progression of the disease, coupled with a lack of a referral system in rural areas, remains one of the significant challenges to the sentinel surveillance strategy. One of the major problems with this sentinel surveillance is the possible non-reporting of cases from distant areas/sub-clinical cases. Although the strategy has been modified to counter this, this surveillance is insufficient to give us an idea of the community prevalence of Nipah in Bangladesh. A population-based serological survey for NiV infection could give us an answer to this hypothesis. Another significant limitation of this surveillance is the unavailability of on-site testing facilities. A real-time testing facility would speed up the overall surveillance process, enable the authority to initiate containment/mitigation measures sooner, and ultimately, give us a better chance at saving lives.

### Conclusion

The information generated through the national Nipah surveillance in Bangladesh has had a significant impact on the national and international efforts to detect and mitigate Nipah virus outbreaks. Concurrently, it has also paved the way for research on NiV transmission, pathogenicity, and disease epidemiology. Based on this surveillance platform, several research studies have been initiated on the Nipah vaccine and therapeutics against human Nipah virus infection. Nevertheless, there are significant scopes and demands for improvement. As no vaccines/therapeutics are available for this infection, steps should be taken to improve the surveillance strategy further, to help with rapid case detection, and to give ourselves a better chance at preventing a large-scale epidemic.
